# Collaborative effort: managing Bardet-Biedl syndrome in pediatric patients. Case series and a literature review

**DOI:** 10.3389/fendo.2024.1424819

**Published:** 2024-07-18

**Authors:** Maria Nowak-Ciołek, Michał Ciołek, Agnieszka Tomaszewska, Friedhelm Hildebrandt, Thomas Kitzler, Konstantin Deutsch, Katharina Lemberg, Shirlee Shril, Maria Szczepańska, Agnieszka Zachurzok

**Affiliations:** ^1^ Students’ Scientific Association at the Department of Pediatrics, Medical University of Silesia in Katowice, Zabrze, Poland; ^2^ Students’ Scientific Association at the Department of Psychiatry and Psychotherapy of Developmental Age, Medical University of Silesia in Katowice, Katowice, Poland; ^3^ Prenatal Diagnostics and Genetic Clinic, Public Hospital No.1, Zabrze, Poland; ^4^ Department of Pediatrics, Boston Children’s Hospital, Harvard Medical School, Boston, MA, United States; ^5^ Department of Pediatrics, Faculty of Medical Sciences, Medical University of Silesia in Katowice, Zabrze, Poland

**Keywords:** Bardet-Biedl syndrome, BBS, obesity, genetics, rare diseases

## Abstract

Bardet-Biedl Syndrome (BBS) is an autosomal recessive non-motile ciliopathy, caused by mutations in more than twenty genes. Their expression leads to the production of BBSome-building proteins or chaperon-like proteins supporting its structure. The prevalence of the disease is estimated at 1: 140,000 – 160,000 of life births. Its main clinical features are retinal dystrophy, polydactyly, obesity, cognitive impairment, hypogonadism, genitourinary malformations, and kidney disease. BBS is characterized by heterogeneous clinical manifestation and the variable onset of signs and symptoms. We present a case series of eight pediatric patients with BBS (6 boys and 2 girls) observed in one clinical center including two pairs of siblings. The patients’ age varies between 2 to 13 years (average age of diagnosis: 22 months). At presentation kidney disorders were observed in seven patients, polydactyly in six patients’ obesity, and psychomotor development delay in two patients. In two patients with kidney disorders, the genetic tests were ordered at the age of 1 and 6 months due to the presence of symptoms suggesting BBS and having an older sibling with the diagnosis of the syndrome. The mutations in the following genes were confirmed: *BBS10, MKKS*, *BBS7/BBS10*, *BBS7*, *BBS9*. All described patients developed symptoms related to the urinary system and kidney-function impairment. Other most common symptoms are polydactyly and obesity. In one patient the obesity class 3 was diagnosed with multiple metabolic disorders. In six patients the developmental delay was diagnosed. The retinopathy was observed only in one, the oldest patient. Despite having the same mutations (siblings) or having mutations in the same gene, the phenotypes of the patients are different. We aimed to addresses gaps in understanding BBS by comparing our data and existing literature through a narrative review. This research includes longitudinal data and explores genotype-phenotype correlations of children with BBS. BBS exhibits diverse clinical features and genetic mutations, making diagnosis challenging despite defined criteria. Same mutations can result in different phenotypes. Children with constellations of polydactyly and/or kidney disorders and/or early-onset obesity should be managed towards BBS. Early diagnosis is crucial for effective monitoring and intervention to manage the multisystemic dysfunctions associated with BBS.

## 1 Introduction

Bardet-Biedl Syndrome (BBS) is an autosomal recessive disease, first described by the French physician Bardet in 1920 (1, 2). The prevalence of the disease is estimated at 1: 140,000 – 160,000 births. Currently, there are 24 genes mutations with a confirmed association with BBS phenotype. Their expression leads to the production of BBSome-building proteins (*BBS1, BBS2, BBS4, BBS5, BBS7, BBS8, BBS9, BBS18* genes) or chaperon-like proteins supporting its structure (*BBS6/MKKS, BBS12, BBS10* genes). BBSome is an octamer responsible for transport within the primary cilia, the dysfunction of which leads to the development of the symptoms (3–5). Based on BBS’s pathomechanism it is classified as a ciliopathy along with other conditions such as Alström’s and Meckel’s syndromes (6–8). Moreover, BBS can be attributed to the ever-expanding group of chaperonopathies - diseases caused by defects in chaperones or proteins resembling their structure (9).

BBS symptoms are divided into primary and secondary. A clinical diagnosis can be made in presence of 4 large symptoms or 3 large and 2 small symptoms (1). Nowadays, the clinical diagnosis should be confirmed with genetic tests. Due to the elevated risk of serious organ damage related to BBS, early diagnosis and monitoring of pediatric patients presenting symptoms of this disease is extremely important.

## 2 Methodology

### 2.1 Patients’ selection process and clinical assessment procedures

Patients included in this study are those with molecularly confirmed mutations in BBS-associated genes, identified through clinic’s record search. We gathered data from patients’ medical history to depict symptom development over the course of time and identify key features and trends. It is worth noting that all patients were referred to our clinic from different medical centers across Silesia. therefore, clinical assessment procedures varied prior to BBS diagnosis. Since the admission to the clinic and the diagnosis all the patients followed our assessment protocol, which included:

### 2.2 Genetic testing

Patients were diagnosed in different years via molecular tests available at the given time. P2, P3, P6 and P7 underwent genetic testing as part of nephronophthisis and ciliopathy studies conducted by Professor Hildebrandt in Boston. P1, P4, P5 and P8 were diagnosed in Genetic Clinic (Medical University of Silesia Hospital No.1, Zabrze) with methods presented in **Table 1**.

**Table 1:** Description of used methods in genetic testing among BBS patients.

Patients’ ID	Genetic testing method
P1	Sanger sequencing of BBS10, 2017
P2	NGS, 178 genes related to NPHP and ciliopathy, confirmation by Sanger sequencing, 2018
P3	NGS, 178 genes related to NPHP and ciliopathy, confirmation by Sanger sequencing, 2020
P4	WES, confirmation by Sanger sequencing, 2017
P5	Sibling of P4, Sanger sequencing of the target variant found previously in the sibling, 2021
P6	WES, confirmation by Sanger sequencing, 2020
P7	WES, analysis of 89 genes related to NPHP, confirmed via Sanger, 2015
P8	NGS, 16 genes related to Bardet-Biedl syndrome, confirmation by Sanger sequencing

### 2.3 Literature review

We employed narrative approach for the literature review and aimed to encompass a comprehensive outline of genotype data pertinent to our patient cohort. Emphasis was placed on elucidating the diverse phenotypic manifestations linked to these genotypes. Special attention was dedicated to exploring the major symptoms and neurological abnormalities, especially their biological pathomechanism in BBS. This approach ensured a thorough understanding of the genotype-phenotype relationships relevant to our research objectives.

## 3 Description of the cases

In this article, we present eight pediatric patients with BBS with the confirmed mutation in BBS-related genes, observed in our clinical center. Each paragraph describes the patients in context of distinctive features of BBS.

### 3.1 Major and minor symptoms of BBS in pediatric patients

In this paragraph, we present the symptoms of BBS as observed in our patient cohort, highlighting the most frequently occurring manifestations (**Table 2**).

**Table 2:** Major and minor symptoms of the Bardet-Biedl syndrome, marked for each patient.

Symptoms	Patient ID/ occurrence of the features in patients							
Major features	P1	P2	P3	P4	P5	P6	P7	P8
Retinal cone-rod dystrophy	+							
Central obesity	+	+	+		+	+		+
Postaxial polydactyly	+	+		+	+	+		+
Cognitive impairment	+		+	+	+	+		
Hypogonadism and genitourinary abnormalities^1^			+		+			
Kidney disease	+	+	+	+	+	+	+	+
Minor features								
Neurologic abnormalities	+			+				+
Olfactory abnormalities								
Oral/dental abnormalities						+		+
Cardiovascular and other thoraco-abdominal abnormalities						+		
Gastrointestinal abnormalities				+				
Endocrine/metabolic abnormalities^2^	+							

P - patient; 1-8 - numbers of the patients; 1 - in males: cryptorchidism, short scrotum, micropenis, and low testicular volume, suggesting the possibility of infertility1; in women - anatomical anomalies found were hypoplastic or duplex uterus, hypoplastic fallopian tubes and/or ovaries, septate vagina, partial or complete vaginal atresia, absent vaginal and/or urethral orifices, hydrocolpos/hydrometrocolpos, persistent urogenital sinus, and vesicovaginal fistula; 2 - metabolic syndrome, subclinical hypothyroidism, type II diabetes (T2DM), polycystic ovary syndrome.

### 3.2 Genotypes

Exact variant details of mutations present in our cohort and their ClinVar interpretatoin are listed in table below (**Table 3**).

**Table 3:** Presentation of genotypes of patients with BBS.

Patient ID	Sex	Gene	Zygosity	Variant details	ClinVar interpretation	Current age	Age of the patient at the time of diagnosis	Clinical reason for genetic testing
P1	Male	*BBS10*	Homozygous	NM_024685.4 (*BBS10*):c.145C>T (p.Arg49Trp)	Pathogenic/ likely pathogenic	10 years 11 months	4 years 6 months	obesity, polydactyly, psychomotor delay
P2	Female	*MKKS*	Homozygous	NM_018848.3 (*MKKS*):c.1436C>G(p.(Ser479*))	Pathogenic	5 years 3 months	8 months	obesity, polydactyly, abnormal image of kidneys on ultrasound
P3	Male	*MKKS*	Homozygous	NM_018848.3(*MKKS*):c.1436C>G(p.(Ser479*))	Pathogenic	3 years 4 months	2 months	abnormal image of kidneys on ultrasound, CKD, polydactyly siblings with BBS
P4	Male	*BBS10*	Heterozygous	NM_024685.4 (*BBS10*):c.424G>A (p.Asp142Asn)	Benign/ Likely benign	13 years 8 months	6 years	obesity, polydactyly, psychomotor delay, ASD
		*BBS7*	Homozygous	NM_176824.3 (*BBS7*):c.1967_1968delinsC (p.Leu656Profs*18)	Pathogenic			
P5	Male	*BBS10*	Heterozygous	NM_024685.4 (*BBS10*):c.424G>A (p.Asp142Asn)	Benign/ likely benign	2 years 11 months	2 months	abnormal image of kidneys on ultrasound, polydactyly, siblings with BBS
		*BBS7*	Homozygous	NM_176824.3 (*BBS7*):c.1967_1968delinsC (p.Leu656Profs*18)	Pathogenic			
P6	Male	*BBS7*	Compound heterozygous	NM_176824.3 (*BBS7*): c.1967_1968delinsC(p.(Leu656Profs*18))	Pathogenic	4 years 7 months	9 months	abnormal image of kidneys on ultrasound, CKD, polydactyly,
P7	Male	*BBS7*	Compound heterozygous	NM_176824.3 (BBS7): c.754G>T(p.(Asp252Tyr))	no data given	7 years 2 months	1 month	abnormal image of kidneys on ultrasound
		*BBS7*	Compound heterozygous	NM_176824.3 (BBS7): c.1967T>C(p.(Leu656Pro))	no data given			
		*BBS7*	Compound heterozygous	NM_176824.3 (BBS7): c.280A>C(p.(Thr94Pro))	no data given			
P8	Female	*BBS9*	Homozygous	NM_001033604.2 (*BBS9*):c.1687C>T	Pathogenic	4 years	2 years 6 months	abnormal image of kidneys on ultrasound, polydactyly

(CKD, chronic kidney disease; ASD, autism spectrum disorder).

### 3.3 Kidney and urinary system

**Figure 1:**
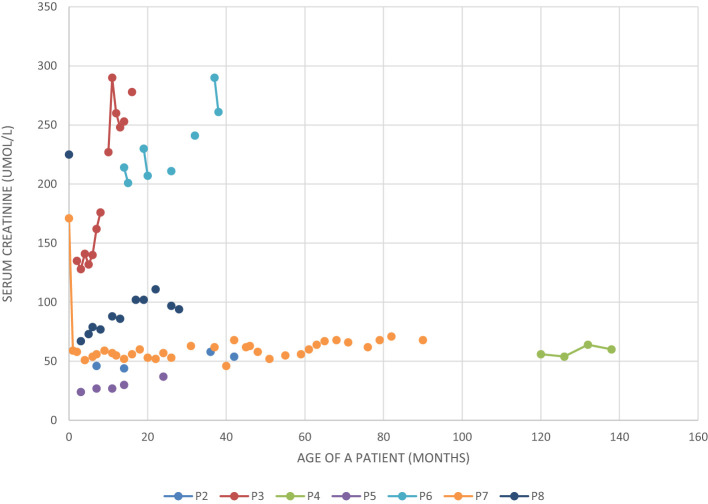
Serum creatinine level in five patients with BBS and chronic kidney disease.

All the described patients developed symptoms related to the kidney and urinary system (**Table 4**). Patient P1 developed only kidney malformations. Seven patients (P2 – P8) developed both kidney malformations and chronic kidney disease (CKD), whereas in six of them those malformations were diagnosed prenatally (P2, P3, P5 – P8). Furthermore, laboratory tests showed increased serum creatinine levels in all our patients at some point of the disease duration, although chronic elevation was observed only in P3 and P6-P8 (**Figure 1**). Patients P3 and P6 require regular dialysis and wait for kidney transplantation.

### 3.4 Obesity

**Figure 2:**
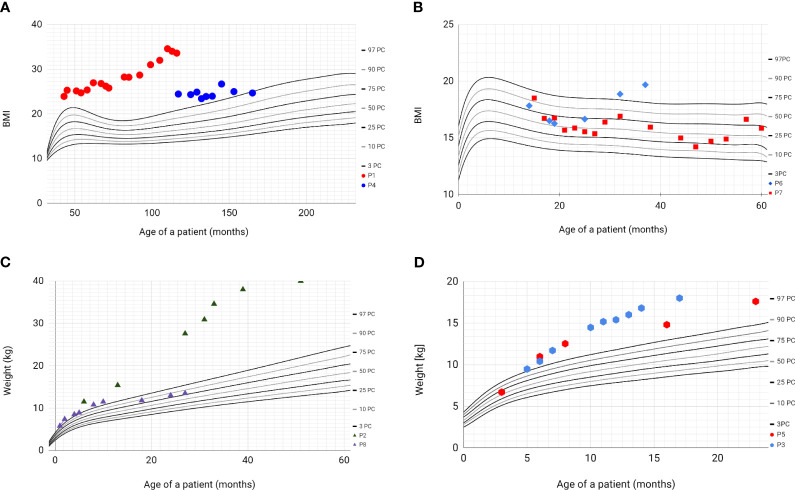
Percentile grids with BMI values and weight values of patients with BBS. Based on WHO Child Growth Standards 2007 WHO Reference. **(A)** - BMI values of patients P1 (red) and P4 (blue); **(B)** - BMI values of patients P6 (blue) and P7 (red); **(C)** - weight values of patients P2 (green) and P8 (purple); **(D)** - weight values of patients P3 (blue) and P5 (red).

All patients’ parents received dietary counselling and lifestyle intervention during each clinical encounter. Based on the history obtained from four patients (P1, P2, P3, P5), hyperphagia and a lack of satiety were observed. Over the course of the follow-up period, only one patient (P7) did not develop obesity, with the highest recorded BMI z-score reaching 0.55. Among these individuals, five had a notable increase in body weight (**Figure 2**). In a solitary instance, represented by patients P4 and P8, a reduction in weight was observed. Notably, patient P1 was classified as having obesity class 3, denoted by a BMI z-score of 4.37, and additionally presented with multiple metabolic comorbidities, including metabolic-associated fatty liver disease (MAFLD), hypertension, insulin resistance, and dyslipidaemia.

Lipid metabolism disorders were confirmed by the results of laboratory tests, including those chronically elevated such as: concentration of total cholesterol (P1, P2, P7), triglycerides (P1, P2, P4, P6, P7), LDL cholesterol fraction (P1, P6). Patients also had elevated activity of alanine aminotransferase (P1) and aspartate aminotransferase (P6, P7).

### 3.5 Polydactyly

Postaxial polydactyly is one of the first symptoms noticed in infants with BBS. In our group, only two patients (P3 and P7) did not have additional toes or fingers. Polydactyly was treated surgically in each case.

### 3.6 Siblings

In our cohort there are two siblings (P2 and P3 - first pair of siblings; P4 and P5 - second pair of siblings).

The first siblings share the same mutation in *MKKS* gene. Although they share the same genotype, their phenotypes differ. The prenatal ultrasound examination was performed on both siblings (P2 and P3). Patient 2 had dilated pelvicalyceal system, whereas Patient 3 had kidney cysts. Furthermore, Patient 2 presents a slower progression of CKD compared to her brother (**Table 4**, **Figure 1**). Both live with obesity, but P2 shows a greater weight gain than her brother (**Figures 2C, D**) Furthermore, the girl (P2) is exhibiting age- appropriate intellectual development, attending a standard preschool program, while her younger brother (P3) presents with delayed psychomotor development. The polydactyly was present in both (**Table 5**). At the moment, P2 presents only 3 large symptoms of BBS, whereas in her younger brother 5 of 6 large symptoms of BBS are observed (**Table 2**).

**Table 4:** Presentation of symptoms of patients with BBS associated with kidney and urinary tract.

Title 1	P1	P2	P3	P4	P5	P6	P7	P8
Age of onset of kidney function impairment	3 years	2 years 8 months	2 months	10 years	1 year 4 months	1 year 3 months	11 months	3 months
CKD	Stage I	Stage II/III	Stage V	Stage I	Stage II	Stage V	Stage III	Stage IV
Prenatal ultrasound examination		dilated pelvicalyceal system	renal cysts		abnormal kidney structure with bilateral hydronephrosis	abnormal structure of the kidneys, bilateral hydronephrosis	dysplasia of both kidneys	fetal polycystic kidneys suspected
Kidney malformation (ultrasound examination) on diagnosis	horseshoe kidney, periodic albuminuria	loss of cortico-medullary differentiation	dilatation of the pelvicalyceal system, (both kidneys), lack of corticomedullary differentiation, cyst in the right kidney	dilatation of the pelvicalyceal system (both kidneys), albuminuria	abnormal structure of both kidneys and dilatation of the pelvicalyceal system	left renal hypoplasia, loss of corticomedullary differentiation	Loss of corticomedullary differentiation, crossed renal ectopia	cysts in both kidneys
Others	hypertension		require regular dialysis, waiting for kidney transplantation			require regular dialysis, waiting for kidney transplantation		hypertension

CKD, chronic kidney disease.

**Table 5:** Presentation of polydactyly in patients with BBS.

Title 1	P1	P2	P3	P4	P5	P6	P7	P8
Accessory VI toe (left foot)	+	+		+		+		+
Accessory VI toe (right foot)	+			+				+
Accessory VI finger on a right hand					+			
Accessory VI finger on a left hand								+
Underdeveloped accessory fingers		+		+				

The second siblings seem to be more difficult to compare due to the greater age difference between two brothers (in siblings 1 the age difference is 2 years, in siblings 2 – 11 years). However, both fulfil the clinical criteria of BBS without any doubt. The patients carry the same homozygous pathogenic mutation in the *BBS7*, as well heterozygous mutation in the *BBS10* gene, identified as benign/likely benign.

The ultrasound examination performed on the older brother (P4) showed a slight asymmetry of the kidneys, enlargement of the pelvicalyceal system, while the prenatal ultra-sound of Patient 5 revealed abnormal kidney structure and enlarged pelvicalyceal system (**Table 4**). They both developed CKD at various stages (P4 – CKD stage I, P5 – CKD stage II). Cognitive impairment was noted in both cases, with a delayed onset of speech observed in P5, and a diagnosis of Autism Spectrum Disorder in P4. Additionally, imaging studies confirmed the partial empty sella syndrome without clinical manifestations.

## 4 Discussion and literature review

BBS is a rare disease; hence, the existing literature may be lacking a thorough picture of this syndrome. Scarce data on the subject makes it difficult to precisely identify gaps in our knowledge. We believe this study can aid this effort as our cohort comprises early-diagnosed children. Although some of the patients share the same genotype, theirs phenotypes vary. Throughout this paper, we compare and investigate those differences. Moreover, our data provides useful insight into mutation-symptom correlation as our cohort represents some unique genetic mosaic incorporating, benign, recessive and pathogenic genes. We gathered longitudinal data on the BBS development in our patients using both prospective and retrospective methods, and we synthesize approaches from different medical faculties. Additionally, we decided to delve into the relationship between phenotype and genotype in BBS, as this topic is currently being explored by other authors as well (10).

We focused on the primary BBS symptoms. The first ones noted based in patients’ history were polydactyly and urinary system disorders. However, these symptoms did not serve as a basis for initiating early diagnosis. The key features that guided physicians toward a BBS diagnosis were polydactyly (in anamnesis), obesity and kidney disease. Regarding the most frequently occurring BBS features, we noted: retinitis pigmentosa (1 patient), obesity (6 patients), kidney disease (8 patients), and developmental delay (6 patients), thus making these features the main topic of this section.

We also observed a few minor features in our patients. Neurological abnormalities were present in three patients (P1, P4, P8). Given their frequent association with cognitive impairment and the abundance of available research data, we summarized these findings in the discussion section. In two patients (P6 and P8) we identified oral abnormalities in the form of micrognathism (P6) and hypoplastic teeth (P8). Furthermore, among the eldest patients, we identified gastrointestinal abnormalities (gastroesophageal reflux disease in P4) and metabolic disturbances (metabolic-associated liver disease, insulin resistance, metabolic syndrome in P1). Due to the limited occurrence of these features in only a few patients, we have decided not to elaborate on them further.

### 4.1 Diagnostic process

According to literature the typical age of the BBS diagnosis varies from 8 to 9 years among pediatric population (11). However, the age of the child at the time of diagnosis may depend on the clinical vigilance of the healthcare provider as well as the main or leading feature of the BBS. The initial symptoms of this syndrome may manifest considerably earlier. Notably, in our study, the average age at which BBS was diagnosed was 22 months [range: 1 month to 6 years], a significantly earlier age, owing to the collaborative efforts of an experienced interdisciplinary team. Numerous prenatal changes can be observed in fetuses with BBS. Among the most frequently described are hyperechogenic kidneys, polydactyly, enlarged kidneys, renal cysts, hydronephrosis and hydrometrocolpos (12–22). However, there is no consensus on which of these features is the most characteristic of BBS.

In a study conducted by Simonini et al., 94.7% of patients with ciliopathies exhibited hyperechoic kidneys, while in fetuses with BBS, the primary renal abnormality was often hydronephrosis. According to this study, genitourinary abnormalities appear to dominate in cases of BBS. Additionally, authors reported that the presence of large hyperechoic kidneys, mimicking sonographic features of infantile polycystic kidney, is a suggestive sign of BBS, a finding also noted by Mary and others (13, 21). When considering only ultrasound (US) detection in fetuses with enlarged and/or renal cysts, the recognition of BBS ranges from 0.3% to 12% (21). In our cohort, kidneys anomalies were present in 6 patients during the prenatal US.

Current literature highlights that the majority of limb abnormalities can be effectively identified in the early stages of pregnancy. This detection of limb abnormalities holds significant clinical importance, given their frequent association with serious underlying disorders, a risk that may be further elevated in fetuses presenting with concurrent abnormalities (12). None of our patients has the prenatal diagnosis of polydactyly.

The most prevalent symptoms detected in our patient cohort at the time of diagnosis included kidney disease, which was present in all individuals, and polydactyly (observed in 6 patients). Subsequently, during follow-up, nearly all patients exhibited excessive body weight and experienced delays in intellectual development (6 out of 8). Additionally, our study revealed that initial symptoms present before BBS diagnosis, such as prenatal manifestations of kidneys and urinary system defects and postnatal occurrences of polydactyly, were observed. It is noteworthy, however, that only a minority of our patients received a diagnosis at such an early stage.

The diagnostic process was significantly shorter among siblings. Based on first siblings’ family history, polycystic kidney disease (ADPKD) and autosomal dominant tubulointerstitial kidney disease (ADTKD) could have been easily excluded, which limited differentiation for the P3. In second siblings, P5 was diagnosed at the age of 4 months according to prenatal US and older brother with BBS. Within our cohort, all patients received BBS confirmation through genetic testing, yet two of them (P2 and P7) did not meet the clinical diagnostic criteria. Nonetheless, this does not preclude the possibility of new symptoms being observed in them in the future. It is possible that P2 and P7 may not exhibit the full BBS phenotype, necessitating a revision of their diagnosis.

### 4.2 Genotype-phenotype association

Bardet-Biedl syndrome is a complex multifactorial disease, which is clearly visible in our cases presentations. The occurrence and severity of symptoms differ based on the developed phenotype which is associated with each mutation. Most of our patients’ phenotype corresponds with their genotype according to the literature (**Table 6**) (3). In P1, with the mutation in *BBS10*, retinopathy manifested early and more metabolic abnormalities were observed compared to other patients. Both patients with mutations in the *BBS7* (P4, P5, P6) and *BBS9* (P8) exhibited a high penetrance of renal anomalies and CKD, characteristics associated with these respective genotypes in the literature. Given the young age of the patients, determining the occurrence of other mutation-associated symptoms requires longer observation.

**Table 6:** Genotype-phenotype association.

Mutated gene	Literature findings	Patient’s symptoms
*BBS6/MKKS*	Manifest a more pronounced clinical phenotype when compared to those with mutations in the *BBS1* gene Deleterious variants display more severe phenotypes (3)	P2: obesity, polydactyly, CKD stage II/III, hypercholesterolemia, hypertriglyceridemia
		P3: obesity, polydactyly, CKD stage V, psychomotor delay, cryptorchidism
*BBS7*	Critical for the function and development of kidneys High penetrance of kidney anomalies (3) Does not affect the expression and function of MCH receptor Accumulation of dopamine receptor (D1R) in cilia (23)	P4: obesity (in the past), polydactyly, CKD stage I, psychomotor delay, ASD, hypertriglyceridemia, astigmatism, myopia
		P5: obesity, polydactyly, CKD stage II, psychomotor delay, micropenis, , astigmatism, myopia
		P6: obesity, polydactyly, CKD stage V, psychomotor delay, micrognathism, hypertriglyceridemia
		P7: CKD stage III, psychomotor delay, hypercholesterolemia, hypertriglyceridemia astigmatism, myopia
*BBS9*	High penetrance of kidney anomalies Critical for the function and development of kidneys (3) Strong correlation between hyperglycemia and insulin resistance and pathogenic variants (24) Large deletion of the *BBS9* gene can lead to the development of a more severe BBS phenotype (e.g., osteopenia with pathological fractures leading to disability, kidney and liver failure, behavioral disorders) and the development of T2DM (10)	P8: obesity (in the past), polydactyly, CKD stage IV, hypertension, hypoplastic teeth
*BBS10*	Manifest a more pronounced clinical phenotype when compared to those with mutations in the *BBS1* gene Deleterious variants display more severe phenotypes (3) Often present with early-onset visual impairment More commonly associated with polydactyly and renal anomalies than their counterparts with mutations in the BBS1 gene (25) Correlate with a higher prevalence of visceral obesity and insulin resistance (26, 27) (ages 2 to 11) higher BMI than children with *BBS1* gene mutation (26) Severe kidney disease is correlated pathogenic variants in BBS10 gene (predominantly truncating variants) (11)	P1: retinal cone-rod dystrophy, obesity, polydactyly, CKD stage I, psychomotor delay, metabolic-associated fatty liver disease, hypertension, insulin resistance hypercholesterolemia, hypertriglyceridaemia

MCH receptor, melanin-concentrating hormone receptors; CKD, chronic kidney disease; ASD, atrial septal defect; T2D, type 2 diabetes mellitus, BMI, body mass index.

### 4.3 Kidneys and urinary system

All of the described patients developed symptoms related to the kidney and urinary system, and, it is worth noting that it may result from the close collaboration between endocrinologists and nephrologists in our medical center, enhancing our vigilance in diagnosing patients with BBS.

Kidney malfunctions and malformations are the major causes of morbidity and mortality in patients with BBS, however, their severity varies depending on the mutation variant (28). In our cohort, renal impairment is associated with higher number of hospitalizations compared to the other consequences of BBS. In the case of renal phenotypes, confirming complete concordance with previously described mutations proves challenging. Two patients with CKD stage V harbor mutations in the *MKKS*/*BBS6* and *BBS7* genes. Mutations in these genes have also been documented in other patients with varying (milder) degrees of renal disease. This observation leads to the hypothesis that not only mutations in specific genes may be associated with these phenotypes but the combination of those mutations and environmental factors. According to Zacchia et al. patients with BBS can be divided into two groups based on the kidney disease occurrence. The first includes patients with kidney and urinary tract malformations diagnosed in early childhood, while the second comprises adult patients with no clear onset symptoms suggesting CKD (29, 30). Confirmation of this hypothesis requires longer-term observation of our patients. However, even now, we can distinguish two groups in our study: patients with rapidly progressing CKD (P2, P3, P5, P6, P7, P8) and those with slowly progressing renal impairment (P1 and P4). In contrast, Cognard et al. suggest that the kidney malfunction in BBS is caused by age-related decreasing of glomeruli function rather than early-onset glomerulopathy. Moreover, the results of animal studies showed that the tissue-specific *BBS10* mutation, confined within the renal epithelium, did not result in renal abnormalities. In 3-month-old mice without obesity, inactivation of *BBS10* had no major effect on renal function (31). This allows us to formulate an additional hypothesis regarding the influence of certain BBS features on others. Our findings may suggest such relationship in our patients. However, we cannot confirm the existence of this dependency, given the influence of factors such as frequent preschool-age infections, which also exacerbated the underlying condition. Given the rarity of the disease and the lack of long-term studies, it is difficult to clearly identify risk factors for the progression of kidney disease in patients with BBS. The conclusions drawn by the researchers emphasize the importance and the need for long-term follow-up of patients with this disease.

### 4.4 Obesity

Central obesity occurs in 89% of patients with Bardet-Biedl syndrome (1). According to Pomeroy et al. infants delivered at term may not exceed the weight of 4000g, however, in more than half obesity may appear even before the age of two and increased weight gain is particularly evident in the preschool age (26). These observations align with the circumstances of our cases. The birth weights of our patients ranged from 3234 to 3850g. Consistently to the data presented in **Figure 2**, excessive body weight was also evident in patients during early childhood.

Obesity is one of the main therapeutic challenges while handling patients with BBS. Those individuals have deficits in the melanocortin-related signaling pathway, responsible for the feeling of satiety, which leads to increased food intake, a phenomenon that was observed in four of our patients as hyperphagia. A hypothesis regarding the aetiology of obesity in individuals with BBS centers on the compromised membrane expression of the leptin receptor (LepRB) within hypothalamic cells. Leptin, exerts its effects by binding to its receptor in the brain, ultimately leading to reduced food intake and heightened energy expenditure. Genetic modifications affecting LepRB can potentially enhance signaling pathways associated with the onset of obesity (32, 33). Given the cause of obesity in BBS, setmelanotide seems to be a good therapeutic option. It is a melanocortin-4 receptor (MC4R) agonist that was initially meant for the treatment of some forms of monogenic obesity. In 2022y it was approved in therapy of BBS as well, based on the results of clinical trial in this matter (34–36). Recent findings of the phase 3 clinical trial suggest that patients diagnosed with BBS experienced a substantial reduction in both weight and BMI z-score after one year of treatment with setmelanotide. Specifically, there was a notable decrease in weight, ranging as high as -7.6% change from baseline in patients aged 18 years and older as well as significant reduction in the BMI z-score by -0.75 points from baseline in patients below the age of 18 (37). We aspire to incorporate this treatment into our treatment regimen for P1, considering the limited efficacy of behavioral interventions in addressing his obesity and its associated complications. The rest of our obese patients are not qualified for setmelanotide treatment due to their young age and/or severe CKD.

Other evidence suggests that the BBSome plays a pivotal role in influencing the sensitivity of neuronal responses to bone morphogenetic protein 8B (BMB-8B). The potential mechanism linking the BBS1 gene to the development of obesity is further substantiated by the observation of impaired central BMP-8B responsiveness in mice harboring a single missense mutation in the BBS1 gene (38).

Increased adipogenesis may be caused by the dysfunction of primary cilia which is crucial in pre-adipocyte differentiation. Marion et al. discovered the occurrence of transient ciliary formation carrying Wnt and Hh receptors during adipogenesis. Inhibition of BBS10 and BBS12 expression weakens ciliogenesis and activates the GSK3 and PPARγ related proadipogenic pathways. Additionally, these studies showed increased fat accumulation and higher leptin levels in adipocytes derived from skin fibroblasts of BBS patients compared to the control sample (39). This data may account for the limited effectiveness of interventions in patient P1, who, despite several years of treatment, did not achieve significant improvement.

The extent of hyperplasia and hypertrophy within adipose tissue is age-dependent and exhibits fluctuations over the lifespan. Notably, there is a rapid occurrence of hyperplasia and hypertrophy during early childhood (0–2 years) and adolescence (12– 18 years). However, as demonstrated in longitudinal and cross-sectional investigations conducted by Knittle et al. and other researchers, there tends to be a relative stabilization of hyperplasia during adulthood (40, 41). This may offer some hope for mitigating adult weight gain in patients. A healthier metabolic profile is indicated by the presence of numerous smaller adipocytes, which correlates with heightened insulin sensitivity, reduced levels of inflammation, and diminished ectopic lipid accumulation (42–44). Conversely, extreme states of hypertrophic obesity are marked by diminished hyperplasia and augmented hypertrophic expansion, thereby exacerbating the onset of obesity-related comorbidities (45). We are concerned about the emergence of such effects in our patients. Combined with the lack of satiety as described earlier, it can lead to the rapid development of complications such as type II diabetes, metabolic syndrome and cardiovascular disease.

### 4.5 Retinal code-rode dystrophy

The most frequently presented symptom of BBS is retinal cone-rod dystrophy, but other dysfunctions are also described such as nyctalopia, which is usually evident by age 7 to 8 (25). Due to the young age of our patients, the examination revealed the presence of visual impairments, primarily myopia and astigmatism. Dystrophy was observed only in P1. Gradual decline in peripheral vision, diminished color discrimination, and deterioration of visual acuity are observable as the condition progresses. In most cases, individuals reach the status of legal blindness during their second or third decade of life (5, 46). Additional ocular phenotypes documented in the literature encompass central cone-rod dystrophy, widespread severe retinal dystrophy, and choroidal dystrophy (25). Initial assessment might pose challenges due to cognitive impairment (diagnosed in 66% of cases) and the age- related lack of compliance in patients. These factors influence parental observations during our patients’ ophthalmological examinations. Clinicians should also consider the possibility of an inverse relationship, i.e. developmental delay as a result of the visual impairment (1).

### 4.6 Cognitive impairment and neurological abnormalities

Findings regarding cognitive performance in individuals diagnosed BBS vary considerably. For instance, among 21 children who were examined, three demonstrated unimpaired IQ, eleven exhibited intellectual disabilities, and ten had mild intellectual impairments (47). Research conducted on a group of adult patients (n = 34, aged 17–53 years) revealed that 26% of them had intellectual disabilities, with one patient even exhibiting an IQ above 120 (48). In a group of our patients, psychomotor delay was observed in six of them. Primarily, it pertained to speech delay (P1, P3, P5, P6,P7) and muscle hypotonia (P3, P6, P7). Additionally, limited studies conducted thus far have documented anxiety, decreased social dominance, and deficiencies in associative learning in BBS2 and 4 knockout mice. These effects are partially attributed to impaired neurogenesis (49–51).

In the research conducted by Rodig and others *BBS6*/*MKKS* and *BBS8*/*TTC8* knockout mice exhibited decreased anxiety levels. Furthermore, they observed reductions in social behavior and alterations in communication. The genotype did not impact learning skills (52). In more recent findings, a mouse model for Ccdc28b, a modifier of BBS, was found to exhibit characteristics associated with obsessive-compulsive behavior and mild social behavior alterations (53).

In our patient cohort, autistic behaviors were observed in two individuals (P1 and P4), encompassing restricted eye contact, speech delay, motor stereotypies and aggressive tendencies. There are several human studies involving patients with BBS, which provide evidence of autistic behavior in this population. A study conducted by Kerr and colleagues revealed autistic traits in 77% of the participants under investigation (54). In a study on BBS patients aged between 2 and 61 years, 80% of participants have shown social deficits (55). Barnett and colleagues’ report similarly noted that externalizing behaviors like aggression were infrequently observed in a group of 21 children diagnosed with BBS. However, issues with social behavior were prevalent. It is worth noting that hearing abnormalities were observed in 2 of our patients (P1 and P6), potentially leading to erroneous conclusions regarding the presence of speech delays and autistic traits, solely arising from hearing impairment. Nonetheless, it remains unclear whether social difficulties in human patients are primarily attributed to the ciliopathy itself or if they are secondary consequences resulting from sensory deficits or social ostracism.

## 5 Conclusions

Bardet-Biedl syndrome encompasses a wide range of clinical features, even within patients who share the same genetic mutations. Common features of BBS may not be considered out of the ordinary nowadays, therefore postponing the diagnosis. Recognizing these diverse clinical presentations is crucial. The constellation of symptoms such as polydactyly and/or kidney disorders and/or early-onset obesity, if present, should trigger the clinicians’ vigilance and consideration of the BBS diagnosis. In some cases, the presence of an affected older sibling can be a key diagnostic indicator, emphasizing the importance of a comprehensive family medical history in diagnosis. We identified mutations in various BBS-related genes, highlighting the genetic complexity of the condition. Furthermore, it revealed that even siblings with identical mutations may have distinct clinical presentations. Our findings stress the importance of early diagnosis and comprehensive care in managing BBS. Despite differences in symptoms presentations, all BBS genotypes lead to disability and multi-systemic dysfunctions. Early diagnosis allows for timely monitoring of patients’ condition, enabling interventions that can prevent or mitigate potential ramifications. Understanding the intricate clinical manifestations and genetic diversity in BBS is essential for better supporting individuals with this rare genetic condition and improving their outcomes.

## Ethics statement

The study was conducted according to the guidelines of the Declaration of Helsinki, and approved by the Ethics Committee of Medical University of Silesia in Katowice (BNW/NWN/0052/KB/88/24, 16.04.2024).

## Author contributions

MN-C: Writing – review & editing, Writing – original draft, Project administration, Conceptualization. MC: Writing – review & editing, Writing – original draft, Conceptualization. AT: Writing – review & editing, Supervision, Investigation. FH: Writing – review & editing, Investigation. TK: Writing – review & editing, Investigation. KD: Writing – review & editing, Investigation. KL: Writing – review & editing, Investigation. SS: Writing – review & editing, Investigation. MS: Writing – review & editing, Writing – original draft, Supervision, Investigation, Conceptualization. AZ: Writing – original draft, Writing – review & editing, Supervision, Project administration, Investigation, Conceptualization.
